# Design of Event-Triggered Fault-Tolerant Control for Stochastic Systems with Time-Delays

**DOI:** 10.3390/s18061929

**Published:** 2018-06-13

**Authors:** Yi Gao, YunJi Li, Li Peng, Junyu Liu

**Affiliations:** 1 Engineering Research Center of Internet of Things Technology Applications Ministry of Education, Jiangnan University, Wuxi 214122, China; gaoy@wxit.edu.cn (Y.G.); 7141905009@vip.jiangnan.edu.cn (Y.L.); 2 Jiangsu Provincial Sensor Network Engineering Technology Research Center, Wuxi Institute of Technology, Wuxi 214121, China; 3 Jiangsu Key Laboratory of IOT Application Technology, Taihu University of Wuxi, Wuxi 214064, China; 4 Dublin Institute of Technology, 19A Lower Kevin Street, Dublin 8, Ireland; d17124121@mydit.ie

**Keywords:** time delays, event-triggered control, robust *H*_∞_ control, mean-square stability

## Abstract

This paper proposes two novel, event-triggered fault-tolerant control strategies for a class of stochastic systems with state delays. The plant is disturbed by a Gaussian process, actuator faults, and unknown disturbances. First, a special case about fault signals that are coupled to the unknown disturbances is discussed, and then a fault-tolerant strategy is designed based on an event condition on system states. Subsequently, a send-on-delta transmission framework is established to deal with the problem of fault-tolerant control strategy against fault signals separated from the external disturbances. Two criteria are provided to design feedback controllers in order to guarantee that the systems are exponentially mean-square stable, and the corresponding $H_{\infty }$-norm disturbance attenuation levels are achieved. Two theorems were obtained by synthesizing the feedback control gains and the desired event conditions in terms of linear matrix inequalities (LMIs). Finally, two numerical examples are provided to illustrate the effectiveness of the proposed theoretical results.

## 1. Introduction

Network control systems have been widely used in many applications such as video surveillance, satellite clusters, offshore platforms, and mobile robotics, among others, because of their advantages of wireless connectivity, efficiency, and flexibility [1,2,3,4]. However, introducing a network into a control loop can cause some problems, especially when the communication bandwidth is limited. It is indicated that only a few system components can obtain communication resources for data exchange simultaneously, which may affect the system performance and even cause system instability. Furthermore, the main constraint of wireless sensor networks is the limited battery life. Normally, it is impractical to replace batteries so the lifetime of the network control systems is equal to its battery life. The best option to lengthen the battery life is to reduce the wireless communication, which is a major source of energy consumption. The disadvantage of traditional periodic communication and control is that even when the output fluctuation is sufficiently small to change the output signal, the measurement value is also transmitted, resulting in a waste of communication and energy resources of battery-based devices [5,6,7].

The propositions of “replacing periodic control with event-triggered control” have been known since 1950s [8,9]. In addition, the interest on event-triggered control was initiated by the paper [10]. The basic idea of event-triggered control is that communication data based on the measured signals (states or outputs) are sent only when the designed conditions of the event-triggered strategy are satisfied, which can reduce unnecessary calculation and transmission, lower the requirement of a communication network system, and achieve a better balance between the control performance and traffic load [11]. This is particularly important when multiple systems use a shared network to communicate. Compared with time-triggered systems, shared networks can support more event-triggered systems [12]. The event-triggered control scheme can save energy resources of battery-based devices, computation resources, and limited network resources as well [13].

In the last decade, event-triggered control has become a hot research topic and significant contributions have been made [14,15,16,17,18,19,20,21,22,23,24,25,26]. The event-triggered data sampling strategies based on send-on-delta have been investigated in [19,20]. Recent discussions of event-triggered control for stochastic systems could be found in [21,22]. Literature [23,24] studied the event-triggered strategy of uncertain systems, and some of them are applicable to nonlinear systems. In addition, the event-triggered strategy for transmission time-delayed systems was studied in literature [25,26]. However, the reliability of the sensors cannot always be guaranteed because of actuator faults and unknown disturbances, the problem of fault-tolerant control has been actively investigated [27,28,29]. Notably, event-triggered fault-tolerant control for stochastic systems with state delays has not been adequately addressed. Thus, the main contributions of this paper can be summarized as follows: two novel event-triggered fault-tolerant control strategies are proposed based on a state and a send-on-delta event generators for a stochastic system with state delays. The closed-loop networked control system is exponentially mean-square stable, and the prescribed $H_{\infty }$ disturbance attenuation performance is also achieved. A simple algorithm is developed to deal with the addressed problem, which can be easily implemented using an efficient linear matrix inequalitie (LMI) toolbox.

This paper is organized as follows. Section 2 formulates the problem and some important lemmas are presented. Our main results are described in Section 3, the state-based event-triggered controls are presented in Section 3.1. Section 3.2 describes the send-on-delta strategy. Two numerical examples are presented in Section 4 to illustrate the results. Section 5 concludes this paper.

*Notations*: The superscript “T” stands for matrices transport. $R^{n}$ and $R^{n\times m}$ denote *n* dimensional Euclidean space and set of all n×m matrices, respectively. For a square matrix *S*, $S>0\left(S<0\right)$ means that this matrix is positive definite (negative definite). In symmetric block matrices, “*” is used as an ellipsis for terms induced by symmetry. *I* denotes an identity matrix with appropriate dimensions. Let (Ω,F,P) be a complete probability space, where Ω is the sample space, F is the σ-algebra of subsets of the sample space, and P is the probability measure on F. Furthermore, E(·) denotes the mathematical expectation of a matrix. $∥.∥$ stands for standard Euclidean norm in $R^{n}$.

## 2. Problem Statement

Consider the following discrete-time linear stochastic system with state delays defined in a probability space (Ω,F,P):

(1) $$\left\{\begin{array}{c} \begin{array}{cc} x(k+1) & =Ax\left(k\right)+A_{d}x\left(k-d\right)+Bu\left(k\right)+\left(A_{2}x\left(k\right)+Dd\left(k\right)+Ff\left(k\right)\right)w\left(k\right)\\ z(k) & =Zx(k) \end{array} \end{array}\right.$$

where *k* is a discrete-time index, $x\left(k\right)\in R^{n}$ is the state vector, $u\left(k\right)\in R^{m}$ denotes the control vector, and x(0) is the initial state. $z\left(k\right)\in R^{p}$ correspond to the controlled output variables. The stochastic variable $w_{k}$ is a scalar Wiener process defined on a complete space (Ω,F,P) with $E\left(w_{k}\right)=0$, $E\left(w_{k}^{2}\right)=1$ and $E\left(w_{i}w_{j}\right)=0$ (i≠j). Moreover, the fault signals f(k) and disturbance signals d(k) are assumed to be $\ell_{2}$ signals ($f,w\in \ell_{2}^{s}$), where *d* is a delay coefficient. The matrices *A*, $A_{2}$, $A_{d}$, *D*, *F* and *Z* are known constant matrices with appropriate dimensions.

**Figure 1:**
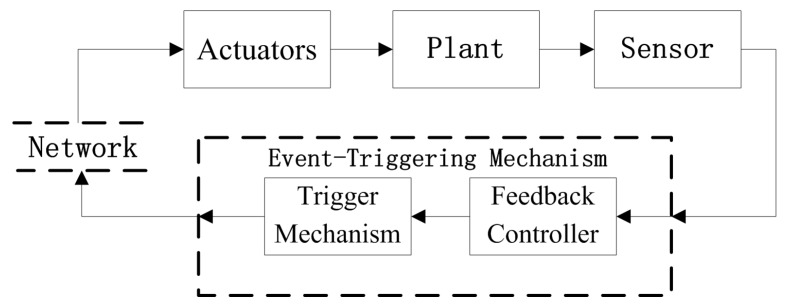
Architecture of the event-triggered network control system.

This paper assumes controllers and sensors to be collocated or hard-wired. The architecture of the event-triggered network control system used in this study is shown in Figure 1, which is similar to literature [7]. The event-triggered mechanism is composed of two units: a feedback controller and a trigger mechanism (event strategy, conditions, or algorithm). The trigger mechanism determines whether the control input should be sent to the actuator via the network. In this event-triggered mechanism, the event condition based on the current controlled output is monitored continuously. Once the condition is satisfied, an event is triggered.

The plant is assumed to be time-driven, whereas the actuator is event-triggered. The actuator is triggered only when a new control vector $u\left(k\right)$ is received. Further, $a\left(k\right)\in \left\{0,1\right\}$ is defined as an event-triggered decision variable that determines whether to send the control vector at each sampling time: when $a\left(k\right)=1$, $u\left(k\right)$ can be calculated and sent out; when $a\left(k\right)=0$, $u\left(k\right)$ cannot be allowed to update. In this study, the event-triggered mechanism and feedback controller are co-designed. The objective is to use the minimum trigger time to maintain the control performance.

**Remark** **1.** The design of the event-triggered strategy must specify the minimum trigger time to avoid the zeno phenomenon, i.e., an infinite number of trigger times in finite time [7]. The system event generator used in this study is time-driven and sampled at a constant frequency; thus, the minimum trigger time of an event-triggered strategy is the sampling time, and hence, no zeno phenomenon can occur.

Before proceeding further, it is necessary to introduce the definition of mean-square stability.

**Definition** **1.** *[30] A discrete stochastic process $\xi_{k}$ is said to be exponentially mean-square stable, if there exist constants $\alpha_{1}>0$ and $0<\alpha_{2}<1$ such that*

(2) $$E\left[{∥\xi_{k}∥}^{2}\right]\leq \alpha_{1}\alpha_{2}^{k}\underset{-d\leq i\leq 0}{\sup}E\left[{∥\xi_{i}∥}^{2}\right],\;\;\;k\in I^{+}$$

*where $I^{+}$ is the set of positive integers and d is a constant.*

With the help of Definition 1, this paper focuses on the co-design of the feedback controller and the event-triggered mechanism such that the discrete-time linear stochastic system (1) satisfies the following requirements simultaneously.

1: When ∂(k)=0, the system is exponentially mean-square stable.
2: Under the zero-initial condition
  (3) $$\sum_{k=0}^{\infty }E\left\{{∥z(k)∥}^{2}\right\}<\gamma^{2}\sum_{k=0}^{\infty }E\left\{{∥\partial (k)∥}^{2}\right\}$$
  for all nonzero ∂(k), where $\partial \left(k\right)=\left[\begin{array}{c} d(k)\\ f(k) \end{array}\right]$, and a γ>0 is prescribed scalar.

Some lemmas are presented, which will play an important role in the proof of our main theorems in Section 3.

**Lemma** **1.** *[31] Suppose Y>0, $x\in R^{n}$ and $w\left(k\right)$ is a Gaussian random vector satisfying $E\left(w\left(k\right)\right)=0$, $E\left(w^{2}\left(k\right)\right)=Q$. Let η be the random variable*

$$\eta ={\left(x+w\right)}^{T}Y\left(x+w\right)$$

then $E\left(\eta \right)=x^{T}Yx+trace\left(Q_{}Y\right)$*.*

**Lemma** **2.** *(S-procedure [32], Lemma 3) Let* $f\left(x\right)$ *and* $g\left(x\right)$ *be two arbitrary quadratic forms over* $R^{n}$*. Then*

$$f\left(x\right)<0\;for\;\forall x\in R^{n}$$

*satisfying* $g\left(x\right)<0$ *if and only if there exist a scalar* τ≥0 *such that* $f\left(x\right)-\tau g\left(x\right)\leq 0$ *for* $\forall x\in R^{n}$*.*

**Lemma** **3.** *[33] Let the matrix* $B\in R^{n\times m}$ *be of full-column rank with singular value decomposition following the structure*

(4) $$B=U^{T}\left[\begin{array}{c} \Sigma \\ 0 \end{array}\right]V^{T}={\left[\begin{array}{c} U_{1}\\ U_{2} \end{array}\right]}^{T}\left[\begin{array}{c} \Sigma \\ 0 \end{array}\right]V^{T}$$

*if there exist positive–definite matrices* $P\in R^{n\times n}$ *satisfying*

(5) $$P=U_{1}^{T}P_{11}U_{1}+U_{2}^{T}P_{22}U_{2}$$

*then there exists an invertible matrix* $M\in R^{m\times m}$ *such that* PB=BM*, where* $M^{-1}=V\Sigma^{-1}P_{11}^{-1}\;\Sigma V^{T}$*.*

## 3. Main Results

In this section, two event-triggered fault-tolerant control strategies are studied based on a state information and a send-on-delta strategy, respectively.

### 3.1. Event-Triggered Control Based on State Information

In this subsection, we intend to design the following event-triggered state feedback controller:

(6) $$u\left(k\right)=\left\{\begin{array}{c} 0\;\;\;\;\;\;\;\;\;\;\;\;\;\;\;\;\;\;\;\;\;\;\;\;\;\;\;\;\;\;\;\;\;\;\;\;\;\;\;a(k)=0\\ Kx(k)\;\;\;\;\;\;\;\;\;\;\;\;\;\;\;\;\;\;\;a(k)=1 \end{array}\right.$$

where *K* represents gain matrices with appropriate dimensions to be determined. Thus, the corresponding closed-loop systems are given by

(7) $$\left\{\begin{array}{c} x\left(k+1\right)=Ax\left(k\right)+A_{d}x\left(k-d\right)+\left(A_{2}x\left(k\right)+Dd\left(k\right)+Ff\left(k\right)\right)w\left(k\right)\;a\left(k\right)=0\\ x\left(k+1\right)=\left(A+BK\right)x\left(k\right)+A_{d}x\left(k-d\right)+\left(A_{2}x\left(k\right)+Dd\left(k\right)+Ff\left(k\right)\right)w\left(k\right)\;a\left(k\right)=1 \end{array}\right.$$

**Remark** **2.** Notably, the fault signals are discussed together with the external disturbance, because they have no influence on the main results; if separated, they will increase the complexity of the following stability analysis.

To design an event-triggered fault-tolerant control strategy and the gain matrices of the controller, the following theorem will be provided to guarantee mean-square stability and the $H_{\infty }$-norm disturbance attenuation level of the closed-loop stochastic system (7).

**Theorem** **1.** *For a given scalar* γ>0*, if there exist real matrices* P>0 *and* Y>0 *satisfying the following matrix inequality:*

(8) $$\left[\begin{array}{cccc} \Omega \;\;\; & {\left(A+BK\right)}^{T}PA_{d} & A_{2}^{T}PD & A_{2}^{T}PF\\ {}* & A_{d}^{T}PA_{d}-Y & 0 & 0\\ {}* & * & D^{T}PD-\gamma^{2} & D^{T}PF\\ {}* & * & * & F^{T}PF-\gamma^{2} \end{array}\right]<0$$

*where* $\Omega ={\left(A+BK\right)}^{T}P\left(A+BK\right)-P+Y+A_{2}^{T}PA_{2}+Z^{T}Z$*.*

*Under the following event-triggering condition:*

(9) $$\left\{\begin{array}{c} \varepsilon^{T}\left(k\right)\left[\begin{array}{cc} A^{T}PA\;\;\; & A^{T}PA_{d}\\ {}* & A_{d}^{T}PA_{d} \end{array}\right]\varepsilon \left(k\right)\leq \varepsilon^{T}\left(k\right)\left[\begin{array}{cc} A_{1}^{T}PA_{1}\;\;\; & A_{1}^{T}PA_{d}\\ {}* & A_{d}^{T}PA_{d} \end{array}\right]\varepsilon \left(k\right)\;a(k)=0\\ otherwise\;a(k)=1 \end{array}\right.$$

*where* $A_{1}=A+BK$ *and* $\varepsilon^{T}\left(k\right)=\left[\begin{array}{cc} x^{T}\left(k\right)\;\;\; & x^{T}\left(k-d\right) \end{array}\right]$*. Then there exists a state-feedback controller K such that the system (7) is exponentially mean-square stable when* ∂(k)=0*. The* $H_{\infty }$*-norm constraint* $\sum_{k=0}^{\infty }E\left\{{∥z(k)∥}^{2}\right\}<\gamma^{2}\sum_{k=0}^{\infty }E\left\{{∥\partial (k)∥}^{2}\right\}$ *is achieved when* ∂(k)≠0*.*

**Proof.**  Consider the following Lyapunov–Krasovskii function:

(10) $$V\left(k\right)=x^{T}\left(k\right)Px\left(k\right)+\sum_{i=k-d}^{k-1}\left(x^{T}\left(i\right)Yx\left(i\right)\right)$$

let $\delta \left(k\right)=A_{2}x\left(k\right)+Dd\left(k\right)+Ff\left(k\right)$, thus, we have

(11) $$\begin{array}{c} E\left[V(k+1)\right]-V\left(k\right)\\ =x^{T}\left(k\right)A_{1}^{T}PA_{1}x\left(k\right)+x^{T}\left(k\right)A_{1}^{T}PA_{d}x\left(k-d\right)+E\left[x^{T}\left(k\right)A_{1}^{T}P\delta \left(k\right)w\left(k\right)\right]\\ +x^{T}\left(k-d\right)A_{d}^{T}PA_{1}x\left(k\right)+x^{T}\left(k-d\right)A_{d}^{T}PA_{d}x\left(k-d\right)+E\left[x^{T}\left(k-d\right)A_{d}^{T}P\delta \left(k\right)w\left(k\right)\right]\\ +E\left[w^{T}\left(k\right)\delta^{T}\left(k\right)PA_{1}x\left(k\right)\right]+E\left[w^{T}\left(k\right)\delta^{T}\left(k\right)PA_{d}x\left(k-d\right)\right]-x^{T}\left(k\right)Px\left(k\right)\\ +E\left[w^{T}\left(k\right)\delta^{T}\left(k\right)P\delta \left(k\right)w\left(k\right)\right]+x^{T}\left(k\right)Yx\left(k\right)-x^{T}\left(k-d\right)Yx\left(k-d\right) \end{array}$$

As $E\left[w(k)\right]=0$, it follows that

(12) $$\begin{array}{c} E\left[x^{T}\left(k\right)A_{1}^{T}P\delta \left(k\right)w\left(k\right)\right]=E\left[x^{T}\left(k-d\right)A_{d}^{T}P\delta \left(k\right)w\left(k\right)\right]=E\left[w^{T}\left(k\right)\delta^{T}\left(k\right)PA_{1}x\left(k\right)\right]\\ =E\left[w^{T}\left(k\right)\delta^{T}\left(k\right)PA_{d}x\left(k-d\right)\right]=0 \end{array}$$

By applying Lemma 1, one can obtain

(13) $$\begin{array}{c} E\left[w^{T}\left(k\right)\delta^{T}\left(k\right)P\delta \left(k\right)w\left(k\right)\right]=trace\left(\delta^{T}\left(k\right)P\delta \left(k\right)\right)=\delta^{T}\left(k\right)P\delta \left(k\right)\\ ={\left(A_{2}x\left(k\right)+Dd\left(k\right)+Ff\left(k\right)\right)}^{T}P\left(A_{2}x\left(k\right)+Dd\left(k\right)+Ff\left(k\right)\right)\\ =x^{T}\left(k\right)A_{2}^{T}PA_{2}x\left(k\right)+x^{T}\left(k\right)A_{2}^{T}PDd\left(k\right)+x^{T}\left(k\right)A_{2}^{T}PFf\left(k\right)\\ +d^{T}\left(k\right)D^{T}PA_{2}x\left(k\right)+d^{T}\left(k\right)D^{T}PDd\left(k\right)+d^{T}\left(k\right)D^{T}PFf\left(k\right)\\ +f^{T}\left(k\right)F^{T}PA_{2}x\left(k\right)+f^{T}\left(k\right)F^{T}PDd\left(k\right)+f^{T}\left(k\right)F^{T}PFf\left(k\right) \end{array}$$

Combining (11), (12) and (13), it follows that

(14) $$\begin{array}{c} E\left[V(k+1)\right]-V\left(k\right)\\ =x^{T}\left(k\right)A_{1}^{T}PA_{1}x\left(k\right)+x^{T}\left(k\right)A_{1}^{T}PA_{d}x\left(k-d\right)+x^{T}\left(k-d\right)A_{d}^{T}PA_{1}x\left(k\right)\\ +x^{T}\left(k-d\right)A_{d}^{T}PA_{d}x\left(k-d\right)+x^{T}\left(k\right)A_{2}^{T}PA_{2}x\left(k\right)+x^{T}\left(k\right)A_{2}^{T}PDd\left(k\right)\\ +x^{T}\left(k\right)A_{2}^{T}PFf\left(k\right)+d^{T}\left(k\right)D^{T}PA_{2}x\left(k\right)+d^{T}\left(k\right)D^{T}PDd\left(k\right)\\ +d^{T}\left(k\right)D^{T}PFf\left(k\right)+f^{T}\left(k\right)F^{T}PA_{2}x\left(k\right)+f^{T}\left(k\right)F^{T}PDd\left(k\right)\\ +f^{T}\left(k\right)F^{T}PFf\left(k\right)-x^{T}\left(k\right)Px\left(k\right)+x^{T}\left(k\right)Yx\left(k\right)-x^{T}\left(k-d\right)Yx\left(k-d\right) \end{array}$$

when ∂(k)=0 and a(k)=1, one can obtain

(15) $$E\left[V(k+1)\right]-V\left(k\right)=\varepsilon^{T}\left(k\right)\left[\begin{array}{cc} A_{1}^{T}PA_{1}-P+Y+A_{2}^{T}PA_{2} & A_{1}^{T}PA_{d}\\ {}* & A_{d}^{T}PA_{d}-Y \end{array}\right]\varepsilon \left(k\right)$$

The condition (8) implies that for $\forall \varepsilon \left(k\right)\in R^{n}$

(16) $$E\left[V(k+1)\right]-V\left(k\right)<0$$

Let

$$F\left[\varepsilon (k)\right]=\varepsilon^{T}\left(k\right)\left[\begin{array}{cc} A_{1}^{T}PA_{1}-P+Y+A_{2}^{T}PA_{2}\;\;\; & A_{1}^{T}PA_{d}\\ {}* & A_{d}^{T}PA_{d}-Y \end{array}\right]\varepsilon \left(k\right)\;G\left[\varepsilon (k)\right]=\varepsilon^{T}\left(k\right)\left[\begin{array}{cc} -I\;\;\; & 0\\ {}* & 0 \end{array}\right]\varepsilon \left(k\right)$$

Thus, we have $F\left[\varepsilon (k)\right]<0$ for $\forall \varepsilon \left(k\right)\in R^{n}$ satisfying $G\left[\varepsilon (k)\right]<0$. According to Lemma 2, it can be demonstrated that there exists a scalar θ>0 such that:

(17) $$F\left(x\right)<\theta G\left(x\right)=\theta \varepsilon^{T}\left(k\right)\left[\begin{array}{cc} -I\;\;\; & 0\\ {}* & 0 \end{array}\right]\varepsilon \left(k\right)=-\theta x^{T}\left(k\right)x\left(k\right)=-\theta {∥x(k)∥}^{2}$$

Thus, it follows that

(18) $$E\left[V(k+1)\right]-V\left(k\right)<-\theta {∥x(k)∥}^{2}$$

Subsequently, by following the proof of Lemma 1 in [30], it can be demonstrated that the system (7) is exponentially mean-square stable when ∂(k)=0 and a(k)=1.

When ∂(k)=0 and a(k)=0, we have $A_{1}=A$. Thus, (14) can be rewritten as

(19) $$\begin{array}{c} E\left[V(k+1)\right]-V\left(k\right)=\varepsilon^{T}\left(k\right)\left[\begin{array}{cc} A^{T}PA-P+Y+A_{2}^{T}PA_{2} & A^{T}PA_{d}\\ {}* & A_{d}^{T}PA_{d}-Y \end{array}\right]\varepsilon \left(k\right)\\ =\varepsilon^{T}\left(k\right)\left[\begin{array}{cc} -P+Y+A_{2}^{T}PA_{2} & 0\\ {}* & -Y \end{array}\right]\varepsilon \left(k\right)+\varepsilon^{T}\left[\begin{array}{cc} A^{T}PA & A^{T}PA_{d}\\ {}* & A_{d}^{T}PA_{d} \end{array}\right]\varepsilon \left(k\right) \end{array}$$

with the event-triggering condition (9), there are some ε(k) satisfying the following inequality, which indicates that when a(k)=0:

(20) $$\varepsilon^{T}\left(k\right)\left[\begin{array}{cc} A^{T}PA\;\;\;\;\;\;\;\; & A^{T}PA_{d}\\ {}* & A_{d}^{T}PA_{d} \end{array}\right]\varepsilon \left(k\right)\leq \varepsilon^{T}\left(k\right)\left[\begin{array}{cc} A_{1}^{T}PA_{1}\;\;\;\;\;\;\;\; & A_{1}^{T}PA_{d}\\ {}* & A_{d}^{T}PA_{d} \end{array}\right]\varepsilon \left(k\right)\;\;\;\;\;$$

Considering (19), one can obtain

(21) $$\begin{array}{c} E\left[V(k+1)\right]-V\left(k\right)\\ \leq \varepsilon^{T}\left(k\right)\left[\begin{array}{cc} -P+Y+A_{2}^{T}PA_{2} & 0\\ {}* & -Y \end{array}\right]\varepsilon \left(k\right)+\varepsilon^{T}\left[\begin{array}{cc} A_{1}^{T}PA_{1} & A_{1}^{T}PA_{d}\\ {}* & A_{d}^{T}PA_{d} \end{array}\right]\varepsilon \left(k\right)\\ =\varepsilon^{T}\left(k\right)\left[\begin{array}{cc} A_{1}^{T}PA_{1}-P+Y+A_{2}^{T}PA_{2} & A_{1}^{T}PA_{d}\\ {}* & A_{d}^{T}PA_{d}-Y \end{array}\right]\varepsilon \left(k\right)<0 \end{array}$$

Similar to the proof of the case of a(k)=1 above, when a(k)=0 one can obtain:

(22) $$E\left[V(k+1)\right]-V\left(k\right)<-\theta {∥x(k)∥}^{2}$$

Thus, the system (7) is exponentially mean-square stable when ∂(k)=0 and a(k)=0.

When ∂(k)≠0 and a(k)=1, let $\varphi^{T}\left(k\right)=\left[\begin{array}{cccc} x^{T}\left(k\right) & x^{T}\left(k-d\right) & d^{T}\left(k\right) & f^{T}\left(k\right) \end{array}\right]$. Considering (14), one can obtain

(23) $$\begin{array}{c} E\left[V(k+1)\right]-V\left(k\right)+z^{T}\left(k\right)z\left(k\right)-\gamma^{2}\partial^{T}\left(k\right)\partial \left(k\right)\\ =\varphi^{T}\left(k\right)\left[\begin{array}{cccc} \Omega & A_{1}^{T}PA_{d} & A_{2}^{T}PD & A_{2}^{T}PF\\ {}* & A_{d}^{T}PA_{d}-Y & 0 & 0\\ {}* & * & D^{T}PD-\gamma^{2} & D^{T}PF\\ {}* & * & * & F^{T}PF-\gamma^{2} \end{array}\right]\varphi \left(k\right) \end{array}$$

The condition (8) implies that

(24) $$E\left[V(k+1)\right]-V\left(k\right)+z^{T}\left(k\right)z\left(k\right)-\gamma^{2}\partial^{T}\left(k\right)\partial \left(k\right)<0$$

Summing up (24) from k=0 to k=∞ results in

(25) $$E\left[V(\infty )\right]-V\left(0\right)+\sum_{k=0}^{\infty }z^{T}\left(k\right)z\left(k\right)-\gamma^{2}\sum_{k=0}^{\infty }\partial^{T}\left(k\right)\partial \left(k\right)<0$$

By the definition of the Lyapunov—Krasovskii function and the zero initial condition, it can be shown that V(∞)>0 and V(0)=0. Thus

(26) $$\sum_{k=0}^{\infty }z^{T}\left(k\right)z\left(k\right)<\gamma^{2}\sum_{k=0}^{\infty }\partial^{T}\left(k\right)\partial \left(k\right)$$

which implies that

(27) $$\sum_{k=0}^{\infty }E\left\{{∥z(k)∥}^{2}\right\}<\gamma^{2}\sum_{k=0}^{\infty }E\left\{{∥\partial (k)∥}^{2}\right\}$$

Hence, the $H_{\infty }$-norm constraint (3) is achieved when ∂(k)≠0 and a(k)=1. Similar to the proof of the case that a(k)=0, it can be shown that the $H_{\infty }$-norm constraint (3) is achieved when ∂(k)≠0 and a(k)=0.

This completes the proof. ☐

Let PB=BM, MK=N, according to the Schur complement, (8) is equivalent to the following LMI

(28) $$\left[\begin{array}{ccccc} -P+Y+A_{2}^{T}PA_{2}+Z^{T}Z & 0 & A_{2}^{T}PD & A_{2}^{T}PF & A^{T}P+N^{T}B^{T}\\ {}* & -Y & 0 & 0 & A_{d}^{T}P\\ {}* & * & D^{T}PD-\gamma^{2} & D^{T}PF & 0\\ {}* & * & * & F^{T}PF-\gamma^{2} & 0\\ {}* & * & * & * & -P \end{array}\right]<0$$

However, equations such as PB=BM cannot be calculated using LMI toolbox of MATLAB. Algorithm 1 based on the Lemma 3 is proposed for obtaining the parameters of (28).
**Algorithm 1** Design procedure for solving the problem of Theorem 1**step 1:** Singular value decomposition of *B* is carried out with the structure of (4) to determine $U_{1}$, $U_{2}$, Σ, and *V*.**step 2:** Replace *P* in (28) with $U_{1}^{T}P_{11}U_{1}+U_{2}^{T}P_{22}U_{2}$.**step 3:** Solve the LMI (28), and obtain $P_{11}$, $P_{22}$, *Q*, *Y*, and *N*.**step 4:** Use the results of steps (1) and (3) to determine $M^{-1}={\left(P_{11}\Sigma V^{T}\right)}^{-1}\Sigma V^{T}=V\Sigma^{-1}P_{11}^{-1}\Sigma V^{T}$.**step 5:** $K=M^{-1}N$.


### 3.2. Event-Triggered Control Based on Send-on-Delta Strategy

Notably, the fault signals are discussed together with the external disturbance in Section 3.1, which is a special case. In this subsection, a send-on-delta strategy is proposed to deal with the fault signals separated from the external disturbance. We are interested in finding the following event-triggered state feedback controller:

(29) $$u\left(k\right)=\left\{\begin{array}{c} u\left(k,l\right)\;a\left(k\right)=0\\ u(k)\;a(k)=1 \end{array}\right.$$

where *K* represents gain matrices with appropriate dimensions to be determined, and the variable $u\left(k,l\right)\;\;\;\;$ represents the latest control input sent to the remote actuator. The control inputs are not periodically sent to the remote actuator owing to the event-triggered data-transmission.

Denoting $\Delta \left(k\right)=u\left(k,l\right)-u\left(k\right)$, the stochastic system (1) via the event-triggered controller (29) can be rewritten as the following close-loop form:

(30) $$x\left(k+1\right)=\left\{\begin{array}{c} A_{1}x\left(k\right)+A_{d}x\left(k-d\right)+\left(A_{2}x\left(k\right)+Dd\left(k\right)\right)w\left(k\right)+Ff\left(k\right)+B\Delta k\;a\left(k\right)=0\\ A_{1}x\left(k\right)+A_{d}x\left(k-d\right)+\left(A_{2}x\left(k\right)+Dd\left(k\right)\right)w\left(k\right)+Ff\left(k\right)\;a\left(k\right)=1 \end{array}\right.$$

where $A_{1}=A+BK$ and *K* represent gain matrices with appropriate dimensions to be designed.

Let us design the feedback gain and the event-triggered control strategy such that the resulting close-loop system (30) is exponentially mean-square stable when ∂(k)=0. The $H_{\infty }$-norm constraint (3) is achieved when ∂(k)≠0.

**Theorem** **2.** *For a given scalar* γ>0 *and* a(k)=0*, if there exist the real matrices* P>0*,* Y>0*,* X>0*,* Q>0*, satisfying the following matrix inequality:*

(31) $$\left[\begin{array}{ccccc} \Theta & A_{1}^{T}PA_{d} & A_{1}^{T}PB & A_{2}^{T}PD & A_{1}^{T}PF\\ {}* & A_{d}^{T}PA_{d}-Y & A_{d}^{T}PB & 0 & A_{d}^{T}PF\\ {}* & * & B^{T}PB-X & 0 & B^{T}PF\\ {}* & * & * & D^{T}PD-\gamma^{2} & 0\\ {}* & * & * & * & F^{T}PF-\gamma^{2} \end{array}\right]<0$$

*where* $\Theta =A_{1}^{T}PA_{1}-P+Y+A_{2}^{T}PA_{2}+Z^{T}Z+Q$*.* *Under the following event-triggering condition:*

(32) $$\left\{\begin{array}{c} \Delta^{T}\left(k\right)X\Delta k-x^{T}\left(k\right)Qx\left(k\right)<0\;a\left(k\right)=0\\ \Delta^{T}\left(k\right)X\Delta k-x^{T}\left(k\right)Qx\left(k\right)\geq 0\;a\left(k\right)=1 \end{array}\right.$$

*Then there exists a state-feedback controller K such that the resulting close-loop system (30) is exponentially mean-square stable when* ∂(k)=0*. The* $H_{\infty }$*-norm constraint* $\sum_{k=0}^{\infty }E\left\{{∥z(k)∥}^{2}\right\}<\gamma^{2}\sum_{k=0}^{\infty }E\left\{{∥\partial (k)∥}^{2}\right\}$ *is achieved when* ∂(k)≠0*.*

**Proof.**  Consider the following Lyapunov–Krasovskii function:

(33) $$V\left(k\right)=x^{T}\left(k\right)Px\left(k\right)+\sum_{i=k-d}^{k-1}\left(x^{T}\left(i\right)Yx\left(i\right)\right)$$

Thus,

(34) $$\begin{array}{c} E\left[V(k+1)\right]-V\left(k\right)\\ =x^{T}\left(k\right)A_{1}^{T}PA_{1}x\left(k\right)+x^{T}\left(k\right)A_{1}^{T}PA_{d}x\left(k-d\right)+x^{T}\left(k\right)A_{1}^{T}PFf\left(k\right)\\ +x^{T}\left(k\right)A_{1}^{T}PB\Delta k+x^{T}\left(k-d\right)A_{d}^{T}PA_{1}x\left(k\right)+x^{T}\left(k-d\right)A_{d}^{T}PA_{d}x\left(k-d\right)\\ +x^{T}\left(k-d\right)A_{d}^{T}PFf\left(k\right)+x^{T}\left(k-d\right)A_{d}^{T}PB\Delta k+x^{T}\left(k\right)A_{2}^{T}PA_{2}x\left(k\right)\\ +x^{T}\left(k\right)A_{2}^{T}PDd\left(k\right)+d^{T}\left(k\right)D^{T}PA_{2}x\left(k\right)+d^{T}\left(k\right)D^{T}PDd\left(k\right)\\ +f^{T}\left(k\right)F^{T}PA_{1}x\left(k\right)+f^{T}\left(k\right)F^{T}PA_{d}x\left(k-d\right)+f^{T}\left(k\right)F^{T}PFf\left(k\right)\\ +f^{T}\left(k\right)F^{T}PB\Delta k+\Delta^{T}\left(k\right)B^{T}PA_{1}x\left(k\right)+\Delta^{T}\left(k\right)B^{T}PA_{d}x\left(k-d\right)\\ +\Delta^{T}\left(k\right)B^{T}PFf\left(k\right)+\Delta^{T}\left(k\right)B^{T}PB\Delta k-x^{T}\left(k\right)Px\left(k\right)+x^{T}\left(k\right)Yx\left(k\right)\\ -x^{T}\left(k-d\right)Yx\left(k-d\right) \end{array}$$

when ∂(k)=0 and a(k)=0, from the event-triggering condition (32), one can obtain

(35) $$x^{T}\left(k\right)Qx\left(k\right)-\Delta^{T}\left(k\right)X\Delta \left(k\right)>0$$

Subsequently, considering $\varphi^{T}\left(k\right)=\left[\begin{array}{ccc} x^{T}\left(k\right) & x^{T}\left(k-d\right) & \Delta^{T}\left(k\right) \end{array}\right]$, and adding (35) into (34) one can obtain

(36) $$E\left[V(k+1)\right]-V\left(k\right)+x^{T}\left(k\right)Qx\left(k\right)-\Delta^{T}\left(k\right)X\Delta \left(k\right)=\varphi^{T}\left(k\right)\Lambda_{1}\varphi \left(k\right)$$

where

(37) $$\Lambda_{1}=\left[\begin{array}{ccc} A_{1}^{T}PA_{1}-P+Y+A_{2}^{T}PA_{2}+Q & A_{1}^{T}PA_{d} & A_{1}^{T}PB\\ {}* & A_{d}^{T}PA_{d}-Y & A_{d}^{T}PB\\ {}* & * & B^{T}PB-X \end{array}\right]$$

The condition (31) implies that $\Lambda_{1}<0$, Considering (35) and (36), it follows that

(38) $$E\left[V(k+1)\right]-V\left(k\right)<0$$

Similar to the proof of Theorem 1 above, it can be shown that the system (30) is exponentially mean-square stable when ∂(k)=0 and a(k)=0.

When ∂(k)≠0 and a(k)=0, considering $\eta^{T}\left(k\right)=\left[\begin{array}{ccccc} x^{T}\left(k\right) & x^{T}\left(k-d\right) & \Delta^{T}\left(k\right) & d^{T}\left(k\right) & f^{T}\left(k\right) \end{array}\right]$, it follows that

(39) $$\begin{array}{c} E\left[V(k+1)\right]-V\left(k\right)+z^{T}\left(k\right)z\left(k\right)-\gamma^{2}\partial^{T}\left(k\right)\partial \left(k\right)+x^{T}\left(k\right)Qx\left(k\right)-\Delta^{T}\left(k\right)X\Delta \left(k\right)\\ =\eta^{T}\left(k\right)\Lambda_{2}\eta \left(k\right) \end{array}$$

where

(40) $$\Lambda_{2}=\left[\begin{array}{ccccc} \Theta & A_{1}^{T}PA_{d} & A_{1}^{T}PB & A_{2}^{T}PD & A_{1}^{T}PF\\ {}* & A_{d}^{T}PA_{d}-Y & A_{d}^{T}PB & 0 & A_{d}^{T}PF\\ {}* & * & B^{T}PB-X & 0 & B^{T}PF\\ {}* & * & * & D^{T}PD-\gamma^{2} & 0\\ {}* & * & * & * & F^{T}PF-\gamma^{2} \end{array}\right]$$

The condition (31) implies that $\Lambda_{2}<0$, thus

(41) $$E\left[V(k+1)\right]-V\left(k\right)+z^{T}\left(k\right)z\left(k\right)-\gamma^{2}\partial^{T}\left(k\right)\partial \left(k\right)<0$$

Similar to the proof of Theorem 1 above, it can be shown that the $H_{\infty }$-norm constraint that $\sum_{k=0}^{\infty }E\left\{{∥z(k)∥}^{2}\right\}<\gamma^{2}\sum_{k=0}^{\infty }E\left\{{∥\partial (k)∥}^{2}\right\}$ of system (30) is achieved when ∂(k)≠0.

Different from Algorithm 1, here we apply the Schur complement for the condition (31) directly. It follows that

(42) $$\left[\begin{array}{cccccccccc} -P_{1} & 0 & 0 & 0 & 0 & P_{1}A^{T}+M^{T}B^{T} & P_{1}A_{2}^{T} & P_{1} & P_{1} & P_{1}\\ {}* & -Y_{1} & 0 & 0 & 0 & Y_{1}A_{d}^{T} & 0 & 0 & 0 & 0\\ {}* & * & -X & 0 & 0 & B^{T} & 0 & 0 & 0 & 0\\ {}* & * & * & -\gamma^{2} & 0 & 0 & D^{T} & 0 & 0 & 0\\ {}* & * & * & * & -\gamma^{2} & F^{T} & 0 & 0 & 0 & 0\\ {}* & * & * & * & * & -P_{1} & 0 & 0 & 0 & 0\\ {}* & * & * & * & * & * & -P_{1} & 0 & 0 & 0\\ {}* & * & * & * & * & * & * & -Q_{1} & 0 & 0\\ {}* & * & * & * & * & * & * & * & -{\left(Z^{T}Z\right)}^{-1} & 0\\ {}* & * & * & * & * & * & * & * & * & -Y_{1} \end{array}\right]$$

where $P_{1}=P^{-1},\;\;Y_{1}=Y^{-1},Q_{1}=Q^{-1},M=KP_{1}\;$. Then, the unknown parameters can be easily calculated with the efficient LMI toolbox of MATLAB.

This completes the proof. ☐

**Remark** **3.** *It is observed that the system (30) is reduced to the following form when* a(k)=1*:* $x_{k+1}=A_{1}x\left(k\right)+A_{d}x\left(k-d\right)+\left(A_{2}x\left(k\right)+Dd\left(k\right)\right)w\left(k\right)+Ff\left(k\right)$*, i.e.,* Δk=0*, and hence, the proof of stability analysis for the case of* a(k)=1 *is omitted.*

**Remark** **4.** *If we change the event-triggering condition (32) to*

(43) $$\left\{\begin{array}{c} \Delta^{T}\left(k\right)X\Delta k-x^{T}\left(k\right)Qx\left(k\right)<\lambda \;a\left(k\right)=0\\ \Delta^{T}\left(k\right)X\Delta k-x^{T}\left(k\right)Qx\left(k\right)\geq \lambda \;a\left(k\right)=1 \end{array}\right.$$

*where* λ>0. *Then the system (30) is no longer exponentially mean-square stable, but exponentially mean-square boundedness.*

## 4. Results and Discussion

In this section, two academic examples are presented to illustrate the properties of the proposed control strategies.

### 4.1. Example 1

Consider system (7) with:

$$A=\left[\begin{array}{cc} 0.9 & 0.5\\ 0.8 & 0.1 \end{array}\right],\;A_{d}=\left[\begin{array}{cc} 0.3 & 0\\ 0.8 & 0.5 \end{array}\right],\;B=\left[\begin{array}{c} 1\\ 0.5 \end{array}\right],\;A_{2}=\left[\begin{array}{cc} 0.1 & 0.3\\ 0.3 & 0.1 \end{array}\right]\;D=\left[\begin{array}{c} 0.15\\ 0.17 \end{array}\right],\;F=\left[\begin{array}{c} -0.5\\ 0.3 \end{array}\right],\;Z=I,\;d=1\;\mathrm{and},\;r=1.6246.$$

which is taken from [34]. By applying the proposed method in Theorem 1 and using the steps of Algorithm 1, the parameters are calculated as follows:

$$P=\left[\begin{array}{cc} 5.5181 & 1.7184\\ 1.7184 & 2.9406 \end{array}\right],\;Y=\left[\begin{array}{cc} 3.4476 & 1.3334\\ 1.3334 & 0.7770 \end{array}\right],\;K=\left[\begin{array}{cc} -1.1703 & -0.3841 \end{array}\right].$$

**Figure 2:**
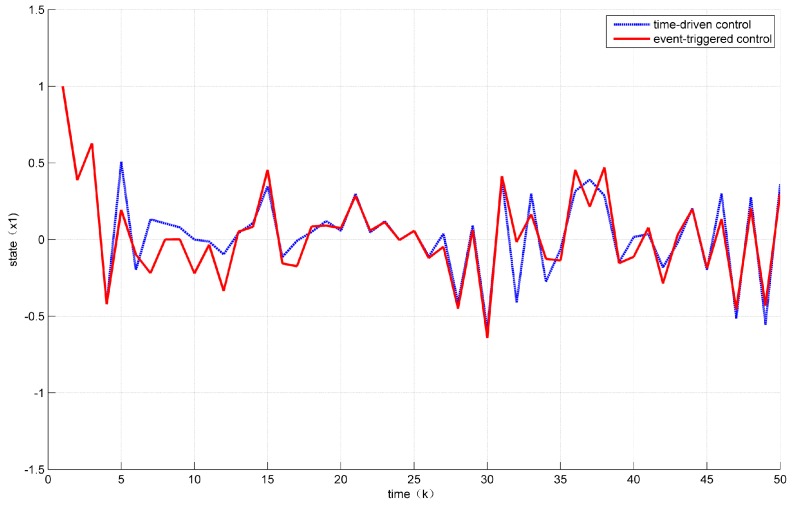
Evolution of state x1.

**Figure 3:**
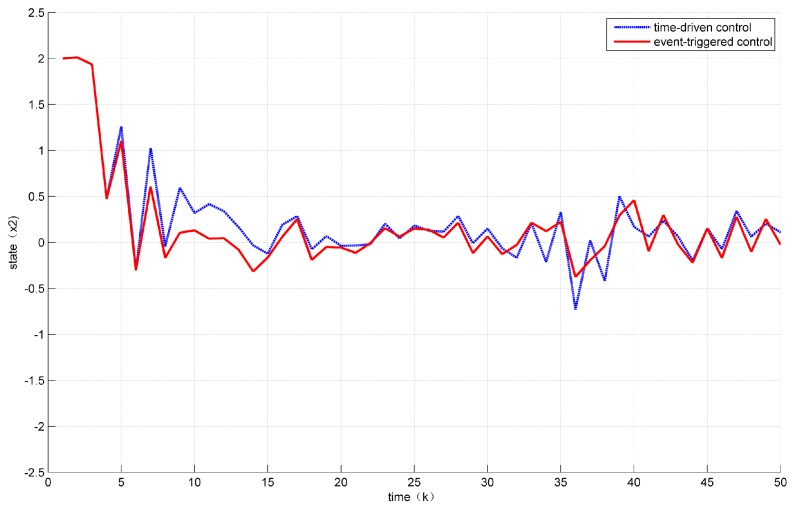
Evolution of state x2.

**Figure 4:**
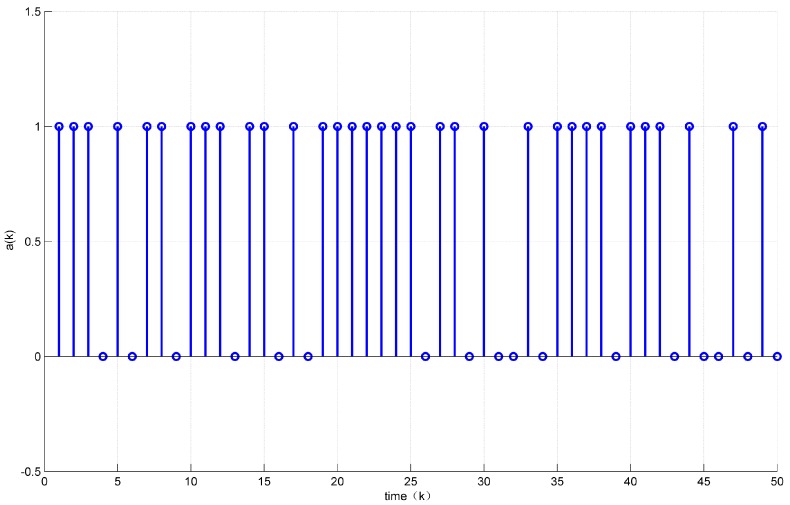
Evolution of a(k).

Assuming the initial condition to be $x\left(k\right)=\left[\begin{array}{c} 1\\ 2 \end{array}\right]$ for k≤0. In addition, the unknown disturbance was selected as $d\left(k\right)=0.2e^{-0.5*k}$, the fault signal is selected as $f\left(k\right)=0.5$. Then, the states’ responses of the system (7) are given in Figure 2 and Figure 3 while the values of a(k) are given in Figure 4.

Figure 4 shows that the control vector u(k) was sent 33 times, i.e., 34% of the network resources were saved.

At the same time, it can be seen from Figure 2 and Figure 3 that the control performance was not much loss compared with the time-driven control.

**Figure 5:**
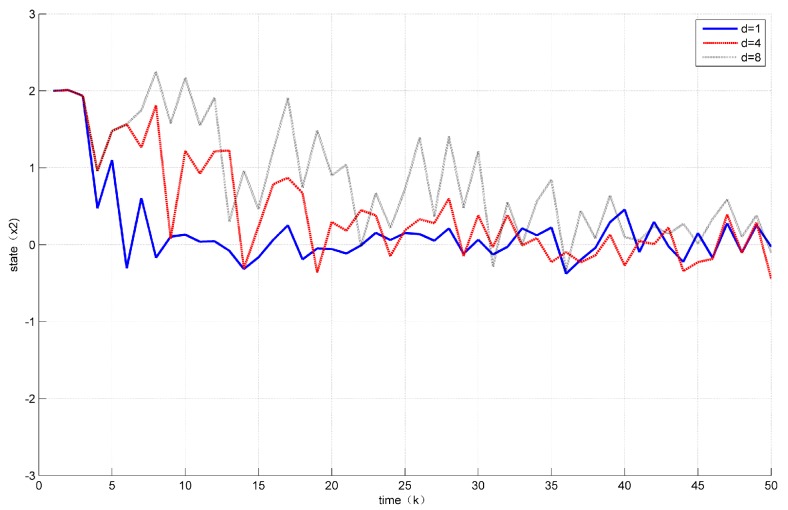
The trajectory of state x2 corresponding to different time delays a(k).

The effect of different time delays on system data transmission and control performance is described below. Figure 5 shows the trajectory of the state corresponding to different time delays. Table 1 shows the number of event triggers corresponding to different time delays.

As can be seen from Figure 5 and Table 1, with the increase of time delay, the convergence speed of the system gradually slows down, the number of event-driven triggers gradually increases, and the system performance gradually deteriorates. When the delay is excessively large for periodic control to guarantee system performance, the event will not be triggered.

### 4.2. Example 2

Consider system (30) with:

$$A=\left[\begin{array}{cc} 0.9951 & 0.2289\\ -0.0177 & 0.8672 \end{array}\right],\;A_{d}=\left[\begin{array}{cc} 0.1 & 0.1\\ 0.1 & 0.1 \end{array}\right],\;B=\left[\begin{array}{cc} -0.4158 & 0.0038\\ 0.0038 & 0.0301 \end{array}\right],\;A_{2}=\left[\begin{array}{cc} 0.1 & 0.3\\ 0.3 & 0.1 \end{array}\right]\;D=\left[\begin{array}{c} -0.15\\ 0.17 \end{array}\right],\;F=\left[\begin{array}{c} -0.2\\ 0.2 \end{array}\right],\;Z=I,\;d=1,\;\lambda =0.1\;\mathrm{and},\;r=2.0061.$$

which is taken from [35]. By applying the proposed method in Theorem 2 and using the LMI toolbox of MATLAB, the parameters were calculated as follows:

$$X=\left[\begin{array}{cc} 3.7139 & 0.0025\\ 0.0025 & 4.3356 \end{array}\right],\;Q=\left[\begin{array}{cc} 0.2471 & -0.0037\\ -0.0037 & 0.2501 \end{array}\right],\;K=\left[\begin{array}{cc} 2.3958 & 0.2869\\ 0.2856 & -28.8468 \end{array}\right].$$

**Figure 6:**
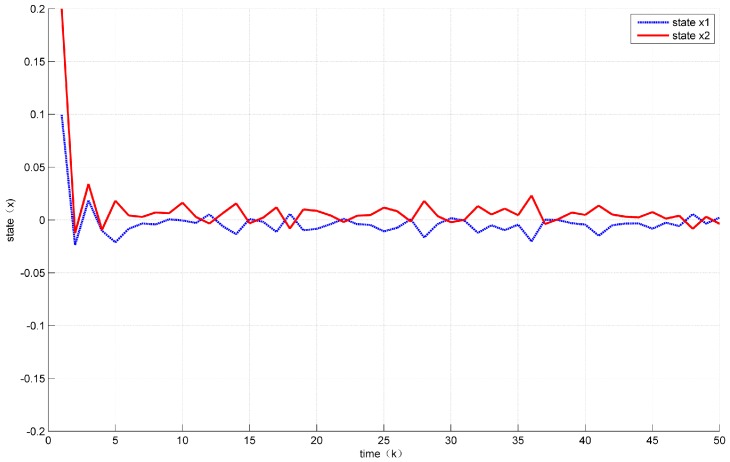
The state trajectories under Theorem 2.

**Figure 7:**
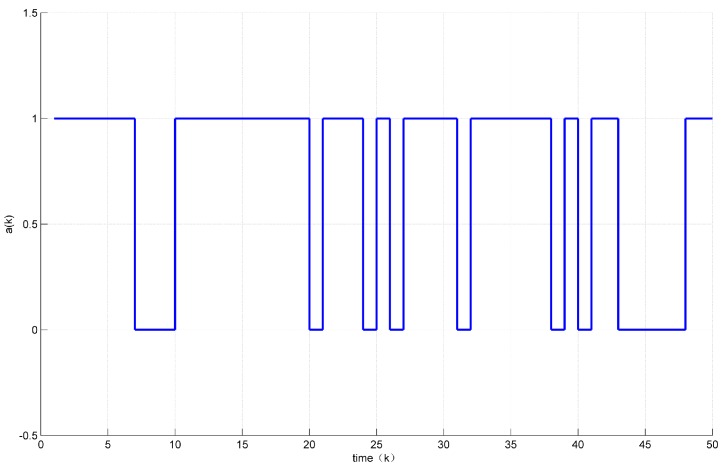
Evolution of a(k).

Assume the initial condition to be $x\left(k\right)=\left[\begin{array}{c} 0.1\\ 0.2 \end{array}\right]$ for k≤0. In addition, the unknown disturbance was selected as $d\left(k\right)=0.02$, the fault signal was selected as $f\left(k\right)=0.05$. The states response of the system (30) are shown in Figure 6 while the values of a(k) are shown in Figure 7.

From the Figures above, we can observe that, compared with time-driven systems, the proposed event-triggered control strategy reduced the unnecessary transmission effectively and found a better balance between the control performance and traffic load.

## 5. Conclusions

In this article, event-triggered fault-tolerant control strategies were investigated for stochastic systems subject to unknown disturbances and state time-delays. To reduce unnecessary calculation and transmission, two novel, event-triggered fault-tolerant control strategies were proposed according to which the systems were exponentially mean-square stable, and the prescribed $H_{\infty }$-norm disturbance attenuation level was achieved. Finally, two academic examples were presented according to which we can conclude that, compared with time-triggered systems, the proposed event-triggered control strategy achieves a better balance between the control performance and traffic load.

## Figures and Tables

**Table 1:** The number of event triggers corresponding to different time delays.

Time Delays	d=1	d=4	d=8
**Trigger Times**	33	40	45

## Abbreviations

The following abbreviations are used in this manuscript:

LD	linear dichroism
IOT	Internet of things
LMIs	linear matrix inequalities
